# The effects of spinal anaesthesia for elective caesarean section on uterine and umbilical arterial pulsatility indexes in normotensive and chronic hypertensive pregnant women: a prospective, longitudinal study

**DOI:** 10.1186/1471-2393-14-291

**Published:** 2014-08-28

**Authors:** Luís Guedes-Martins, Helena Graça, Joaquim P Saraiva, Luísa Guedes, Rita Gaio, Ana S Cerdeira, Filipe Macedo, Henrique Almeida

**Affiliations:** Departamento da Mulher e da Medicina Reprodutiva, Centro Hospitalar do Porto EPE, Largo Prof. Abel Salazar, 4099-001 Porto, Portugal; Department of Experimental Biology, Faculty of Medicine, University of Porto, 4200-319 Porto, Portugal; Department of Anaesthesiology, Centro Hospitalar do Porto EPE, Largo Prof. Abel Salazar, 4099-001 Porto, Portugal; Obstetrics-Gyneacology, Private Hospital Trofa, 4785-409 Trofa, Portugal; Department of Anaesthesiology, Centro Hospitalar S. João, 4200-319 Porto, Portugal; Department of Mathematics, Faculty of Sciences, University of Porto, Porto, Portugal; CMUP-Centre of Mathematics, University of Porto, Porto, Portugal; Gulbenkian Program for Advanced Medical Education, 1067-001 Lisbon, Portugal; Department of Medicine, Beth Israel Deaconess Medical Center, Harvard Medical School, Boston, MA 02215 USA; Department of Medicine, Faculty of Medicine, University of Porto, 4200-319 Porto, Portugal; Serviço de Cardiologia, Centro Hospitalar S. João, 4200-319 Porto, Portugal; Obstetrics-Gynecology, Hospital-CUF Porto, 4100 180 Porto, Portugal

**Keywords:** Spinal anaesthesia, Caesarean section, Blood flow velocity, Hypertension

## Abstract

**Background:**

Despite the known effects of neuraxial blockade on major vessel function and the rapid decrease in uterine vascular impedance, it is unclear how the blockade affects the utero-placental circulation in the near-term. We hypothesize that among women with chronic hypertension, a loss of sympathetic tonus consequent to spinal block may cause significant changes in the utero-placental haemodynamics than the changes typical in normal pregnant women. Therefore, the main study objective was to analyse the effect of spinal anaesthesia for caesarean section on uterine and umbilical arterial impedance in pregnant women at term diagnosed with stage-1 chronic hypertension.

**Methods:**

A prospective, longitudinal study was performed in singleton pregnant women (203 low-risk and 33 with hypertension) scheduled to undergo elective caesarean section. The mean arterial blood pressure and pulsatility indexes for the uterine and umbilical arteries were recorded before and after spinal anaesthesia was performed using 8–9 mg hyperbaric bupivacaine (5 mg/mL) and 2–2.5 μg sufentanil (5 μg/mL). Multiple linear regression models with errors capable of correlation or with unequal variances were fitted using the generalized least squares.

**Results:**

In normotensive women, the mean arterial blood pressure decreased after administering spinal anaesthesia (p < 0.05). The pulsatility index of the uterine and umbilical arteries did not change after spinal anaesthesia. In the hypertensive women, the mean arterial blood pressure (p < 0.05) and uterine artery pulsatility index (p < 0.05) decreased. In both groups, the umbilical artery pulsatility index did not change after spinal anaesthesia.

**Conclusions:**

In stage-1 chronic hypertensive pregnant women at term, spinal anaesthesia for caesarean section reduces uterine artery impedance but not umbilical artery impedance.

**Electronic supplementary material:**

The online version of this article (doi:10.1186/1471-2393-14-291) contains supplementary material, which is available to authorized users.

## Background

Hypertension affects 5–15% of pregnancies [1] and creates additional challenges for the mother [2, 3] and foetus [4, 5]. One major challenge is the increased risk of preeclampsia, eclampsia [6, 7], and related conditions, such as preterm delivery [5, 6] and intrauterine growth restriction [6, 8], that increase morbidity and mortality in the mother [2] and foetus [4, 9].

Introduction of a local anaesthetic, such as bupivacaine, into the subarachnoid space to induce anaesthesia and analgesia has long been used during delivery [10] and is advantaged by a short procedure time [11], rapid onset [10], and high success rate [10, 12]. However, maternal hypotension may develop soon after anaesthetic administration [13–17] as a consequence of sympathetic blockade, which causes arterial and arteriolar vasodilation [18, 19]. Venodilation may also occur, which decreases the cardiac preload and cardiac output, and promotes bradycardia and maternal hypotension. These impacts are further aggravated by aortocaval compression caused by the gravid uterus [15].

Despite the known effects of neuraxial blockade on major vessel function and the rapid decrease in uterine vascular impedance, which can occur in seconds, it is unclear how the blockade affects the utero-placental circulation in the near-term. Furthermore, it is unknown whether hypertension poses additional constraint on local circulatory regulation, because few, if any, studies have evaluated the maternal and foetal haemodynamics in pregnant women with chronic arterial hypertension undergoing spinal anaesthesia.

Many pathophysiologic factors have been implicated in the genesis of chronic arterial hypertension. In most cases, the root cause of the disease remains unknown, but there is mounting evidence that chronic hypertension is initiated and maintained by an elevated sympathetic tone [20]. We hypothesize that among women with chronic hypertension, a loss of sympathetic tonus consequent to spinal block may cause significant changes in the utero-placental haemodynamics than the changes typical in normal pregnant women. Therefore, the current study aimed to compare the effects of spinal anaesthesia comprising hyperbaric bupivacaine for elective caesarean section on uterine and umbilical arteries impedance in normotensive and chronic hypertensive women.

## Methods

### Subjects

This study was approved by the local ethics committee of Centro Hospitalar do Porto–Unidade Maternidade Júlio Dinis. All subjects provided informed consent (IRB protocol number: 133/10 [086-DEFI/126-CES]).

The study was performed from January 2010 to December 2012. Inclusion criteria were: parturient with singleton term pregnancies and gestational age ≥ 37 weeks, healthy condition or stable chronic arterial hypertension without known target organ involvement, scheduled for elective caesarean section due to foetal breech presentation, suspected cephalopelvic disproportion, or previous caesarean.

Exclusion criteria were: patients in labour or with ruptured membranes; those with multiple gestations, coagulopathy, diabetes, or any pregnancy-induced hypertension including preeclampsia; and those receiving β-tocolytic drugs. Subjects were also excluded from the study as follows: contraindication or impossibility to perform spinal anaesthesia, bilateral sensory block that could not be extended to the T6-T4 level, intravenous drug administration that may alter the haemodynamic state (ex. opioids, propofol), or a history of hypersensitivity to local anaesthetic.

Chronic arterial hypertension was defined as a blood pressure of 140/90 mmHg on more on two occasions before 20 weeks of gestation or after 20 weeks of pregnancy if the findings persist beyond 12 weeks postpartum [21]. However, only women with a history of chronic arterial hypertension prior to pregnancy were enrolled in the study. Acceptable medication was folic acid, vitamin, and iron supplements. All hypertensive pregnant women received acetylsalicylic acid (100 mg daily) and methyldopa (750 mg daily). Gestational age was calculated by the crown-rump length between 11 and 14 weeks [22] by several experienced sonographers from the Prenatal Diagnosis Department of our institution.

On the day of caesarean section, biometrical data was collected and the patients were observed by a senior specialist who reviewed their medical record.

The infant was examined by a neonatologist at birth and 1 month later. The new born physical assessment included: Apgar scoring, birth weight, head circumference, abdominal circumference, length, vital signs (temperature, pulse, and respiratory rate), general appearance, and physical and neuromuscular maturity.

The maternal haemodynamic assessment was performed at two time points: (1) before spinal anaesthesia and (2) immediately after inducing spinal anaesthesia. The upper level of dermatome block between T4-T6 was tested by cold sensation and was deemed adequate for surgical anaesthesia.

### Spinal anaesthesia for elective caesarean section

Women were administered intravenous 10 mg metoclopramide and 50 mg ranitidine 2 hours preoperatively. Intravenous cefazoline (2 g) was also administered to all patients 30 minutes before anaesthetic induction. All patients received 500 mL of Lactated Ringer’s solution intravenously 15 minutes before receiving anaesthesia and oxygen (3 L/min) through nasal prongs; blood pressure, electrocardiography, and pulse oximetry were monitored throughout. Patients were placed in a sitting position, the midline approach was used in all women, and the block was performed at the mid-lumbar level. The subarachnoid block was performed in the surgical suite using a combined needle-through-needle spinal-epidural technique (typical for caesarean section at our institution) with an epidural 18-G tuohy needle and 27-G subarachnoid pencil point needle. Spinal anaesthesia comprised 8–9 mg of hyperbaric bupivacaine (5 mg/mL) and 2–2.5 μg sufentanil (5 μg/mL) administered intrathecally, targeting the T4-S4 dermatomes. If the bilateral sensory block could not be extended to the T6-T4 level, then the patient was excluded.

### Blood pressure assessment

Blood pressure (BP) was recorded using an automated instrument (GE Healthcare Carescape™ V100 Vital Signs Monitor with DINAMAP Blood Pressure) at 2-min intervals from induction to delivery. Baseline BP was obtained as the mean of three consecutive measurements taken 2 minutes apart before subarachnoid block and insertion of the epidural catheter; a second measurement was recorded immediately after instilling spinal anaesthesia. The blood pressure was expressed as the mean arterial pressure (MAP) employing the formula:

Maternal hypotension was treated with ephedrine when necessary. The standard for vasopressor (VP) administration was defined as a 20% decrease from the baseline mean arterial pressure at any time after induction. Cases that were adequately managed by increasing the rate of fluid infusion without need for VP administration at the second blood pressure measurement were included. Cases requiring VP administration before the second Doppler flow assessment were excluded.

### Doppler flow assessment

The Doppler flow was evaluated in the right and left uterine and umbilical arteries immediately before (first time point) and after (second time point) spinal anaesthesia using a 4 MHz convex transabdominal probe (GE Healthcare Technologies, GE LOGIC 6, USA). All measurements were made by a single investigator with a high level of expertise in Doppler ultrasound (L.G-M) to minimize inter-observer variability. Intra-observer reliability was estimated from two consecutive readings among the first 30 recordings of the pulsatility indexes in the uterine and umbilical arteries. For uterine artery evaluation, the probe was placed on the lower abdominal quadrants and angled medially, and colour Doppler imaging was used to localize the uterine artery (UtA) as it crossed over the external iliac artery. In all cases, an angle less than 30° was assured before the pulsed Doppler probe was placed over the entire vessel width. Angle correction was then applied, and the signal updated until three similar consecutive waveforms were evidenced to calculate the left and right uterine arteries pulsatility (UtA-PI) indexes as follows:


using the device software (GE Healthcare Technologies, GE LOGIC 6, USA). The mean UtA-PI in the left and right arteries were then determined.

The umbilical artery (U) Doppler flow spectrum was recorded from a free cord loop, and the mean of three consecutive waveforms was analysed to determine the U-PI. Women with absent or reversed diastolic umbilical artery flow before undergoing the subarachnoid block were not included.

No more than 2 minutes were required for the waveform acquisitions. Additionally, the sequence of measurements was extremely accurate and always performed in the same order: (1) right uterine artery, (2) left uterine artery, and finally, (3) the umbilical artery.

### Statistical analysis

Univariate data analysis comprised standard statistical methods: Chi-square test or Fisher’s test (as appropriate) to compare frequencies within a single categorical variable or to determine independence among two factors; and the *t*-test to assess statistically significant differences between means in two independent populations.

Multiple linear regression models with errors that were correlated or had unequal variances were fitted using the generalized least squares and maximum likelihood estimation. Time was considered a dichotomous variable, reflecting the two evaluation time points.

The PI value at vessel *v* (UtA or U), time *t* (before or after spinal anaesthesia), or in a hypertensive patient *h* (hypertensive or normotensive) was denoted by PI (*ν*, *t*, *h*). Dummy variables were considered for the vessels, time, and hypertensive status; the UtA, the time before spinal anaesthesia, and the normotensive status, respectively, were designated as reference categories. The fitted model was as follows:1

with errors *ϵ* following a normal distribution with a zero mean, a variance-covariance matrix having a correlation structure of compound symmetry at the 2-level grouping structure according to the subject or vessel, and different variances according to the vessel. In the above formula, the intercept coefficient *β*_*0*_ and the time-slope coefficient *β*_*1*_ are the linear functions of the vessel, the hypertensive status, and their two-way interaction.

For the chosen MAP model, we obtained the following equation:
2

with errors ϵ following a normal distribution with a zero mean, a variance-covariance matrix with a correlation structure of compound symmetry for each woman, and different variances according to time. The intercept coefficient *β*_*0*_ and the time-slope coefficient *β*_*1*_ were linear functions of the hypertensive status *h*. Dummy variables for the hypertensive status and time were chosen as in model (1).

Data were plotted to assess the assumptions of normality and heteroscedasticity of the above models. The normal plots of the residuals indicated departures from normality in model (1) and no compromising features in model (2). After removing the observations with standardized residuals in the PI model higher than 3.5 (corresponding to 17 observations from 10 different women, with only one hypertensive), the normality problems seemed to resolve. The outliers corresponded to 17 observations from 10 different women, with only woman hypertensive, and essentially matched the women with PI-values higher than the empirical 98^th^ percentile. Graphical analyses confirmed that the variance function models were successful in accommodating the error heteroscedasticity.

Intraclass correlation coefficients (ICC) and 95% confidence intervals were calculated using a two-way mixed-effects model with absolute agreement. ICC was used to assess repeatability because there is sufficient scientific consensus designating an ICC > 0.7 as reflecting a very low measurement error [23, 24]. The reliability coefficient, which is the difference value that will be exceeded by only 5% of measurement pairs on a single subject, was calculated as follows: 1.96 times the standard deviation of the difference between pairs of repeated measurements [23].

Final regression models were chosen based on the lowest Akaike Information Criterion. All statistical analyses were performed using the R language and software environment for statistical computation, version 2.12.1 [25]. The significance level was fixed at 0.05.

Our research has adhered to the Strengthening the Reporting of Observational studies in Epidemiology (STROBE) guidelines for observational studies, and all recommendations were included in the study [Additional file 1].

## Results

A total 277 pregnant women at term were considered eligible for this study according to the established inclusion criteria. Forty-one were excluded (13.9%): 11 patients were in early labour; 8 had multiple pregnancies; 7 had diabetes; 5 required bolus administration of VP prior to the Doppler flow assessment; 4 presented technical difficulties in calculating the pulsatility index of the uterine and umbilical arteries; 3 did not have an adequate anaesthetic blockade; 2 had suspected preeclampsia; and 1 refused to participate in the study.

Among the 236 women enrolled in the study, 203 (86%) were normotensive (NT), and 33 (14%) had chronic arterial hypertension stage 1 (HT).

The main characteristics and pregnancy outcomes of the 236 women are depicted in Table 1. Their ages ranged from 18 to 43 years old; 74% of the subjects were less than 34 years old, and a similar proportion (77%) were not educated beyond the secondary level (maximum of 12 years). For 68% of the women, this was their first pregnancy and most was non-smokers. The mean gestational age at the time of evaluation (caesarean section) was 40.0 weeks (range: 37.0–41.1 weeks). The NT and HT groups (Table 1) showed statistically significant differences in parity (nulliparous predominantly in the HT group), BMI distribution (highest values predominantly in the HT group, p < 0.001), and gestational age at delivery (mean gestational age of HT group was slightly but significantly lower than the NT group, 39.5 vs. 40.1 weeks, p < 0.001). The mean new born weight was 3140.2 g (±340.5 g), with no significant difference between the NT and HT groups (p = 0.059).

**Table 1:** **Demographic characteristics and pregnancy outcomes of the 236 included women**

		All	Normotensive	Hypertensive	
		n = 236	n = 203	n = 33	p*
Age, mean (SD)		30.0 (6.1)	29.7 (6.0)	31.7 (6.5)	0.110
Parity, median (IQR)		0 (0–1)	0 (0–1)	0 (0–0)	0.047
Education level (in years), n(%)	<7	7 (3%)	4 (2%)	3 (9%)	0.199
	7-9	77 (33%)	66 (33%)	11 (33%)	
	10-12	105 (44%)	92 (45%)	13 (40%)	
	>12	47 (20%)	41 (20%)	6 (18%)	
Body Mass Index^a^ (Kg/m), mean (SD)		28.5 (5.6)	27.9 (5.3)	32.5 (5.3)	<0.001
Smoking, n(%)		23 (10%)	19 (9%)	4 (12%)	0.857
Gestational age at delivery (weeks), mean(SD)		40.0 (0.8)	40.1 (0.8)	39.5 (0.8)	<0.001
UtA bilateral notching absence^a^		228 (97%)	196(97%)	32(97%)	1.000
Birth weight at delivery (g), mean (SD)		3140.2 (340.5)	3123.0 (338.8)	3245.9 (337.1)	0.059
Apgar Index 5’	<7	0(0)	0(0)	0(0)	

^a^Body Mass Index and UtA notching were determined before the caesarean section; UtA, uterine artery; SD, standard deviation; IQR, Inter-quartile range. The Chi-square or Fisher’s test were used as appropriate to compare frequencies within a single categorical variable or to determine independence when applied to two categorical variables; the *t*-test was used to compare the means between two independent populations. *p, indicates the homogeneity in the proportions between the hypertensive and normotensive groups.

In the majority of patients (97%), the uterine artery notch was absent bilaterally.

Blood pressure data are presented in Table 2. Hypertensive pregnant women exhibited a significantly higher MAP at both time points (p < 0.001).

**Table 2:** **Mean (SD) MAP of 236 women before and after bupivacaine anaesthetic blockade**

T6-T4 anaesthetic blockade	All (n = 236)	Normotensive (n = 203)	Hypertensive (n = 33)	p
MAP before	86.3 (12.6)	83.1 (10.1)	105.5 (8.9)	<0.001
MAP after	78.8 (8.5)	76.2 (5.7)	94.5 (5.8)	<0.001

SD, standard deviation; MAP, mean arterial pressure.

The reliability coefficients for the UtA-PI and U-PI indexes were 0.111 and 0.076, respectively. The ICC indicating the intra-observer reliability for the UtA-PI and U-PI measurements were very high at 0.963 and 0.899, respectively; the ICC values ranged from 0.923 to 0.982 and 0.797 to 0.951, respectively.

UtA-PI and U-PI data before and after spinal anaesthesia are presented in Table 3. Before anaesthetic blockade, the UtA-PI was significantly higher in the chronic hypertensive pregnant women than in the normotensive patients (p = 0.022).

**Table 3:** **Mean (SD) uterine/umbilical artery PI indexes measured by transabdominal ultrasound before and after bupivacaine anaesthesia**

T6-T4 anaesthetic blockade	UtA-PI				U-PI	
	Normotensive	Hypertensive	p	Normotensive	Hypertensive	p
Before	0.80 (0.20)	0.87 (0.16)	0.022	0.81 (0.10)	0.82 (0.12)	0.596
After	0.78 (0.19)	0.73 (0.15)	0.068	0.81 (0.08)	0.83 (0.12)	0.299

SD, standard deviation; PI, pulsatility index; UtA, uterine artery; U, umbilical artery.

### Multiple analysis

#### MAP model

**Figure 1:**
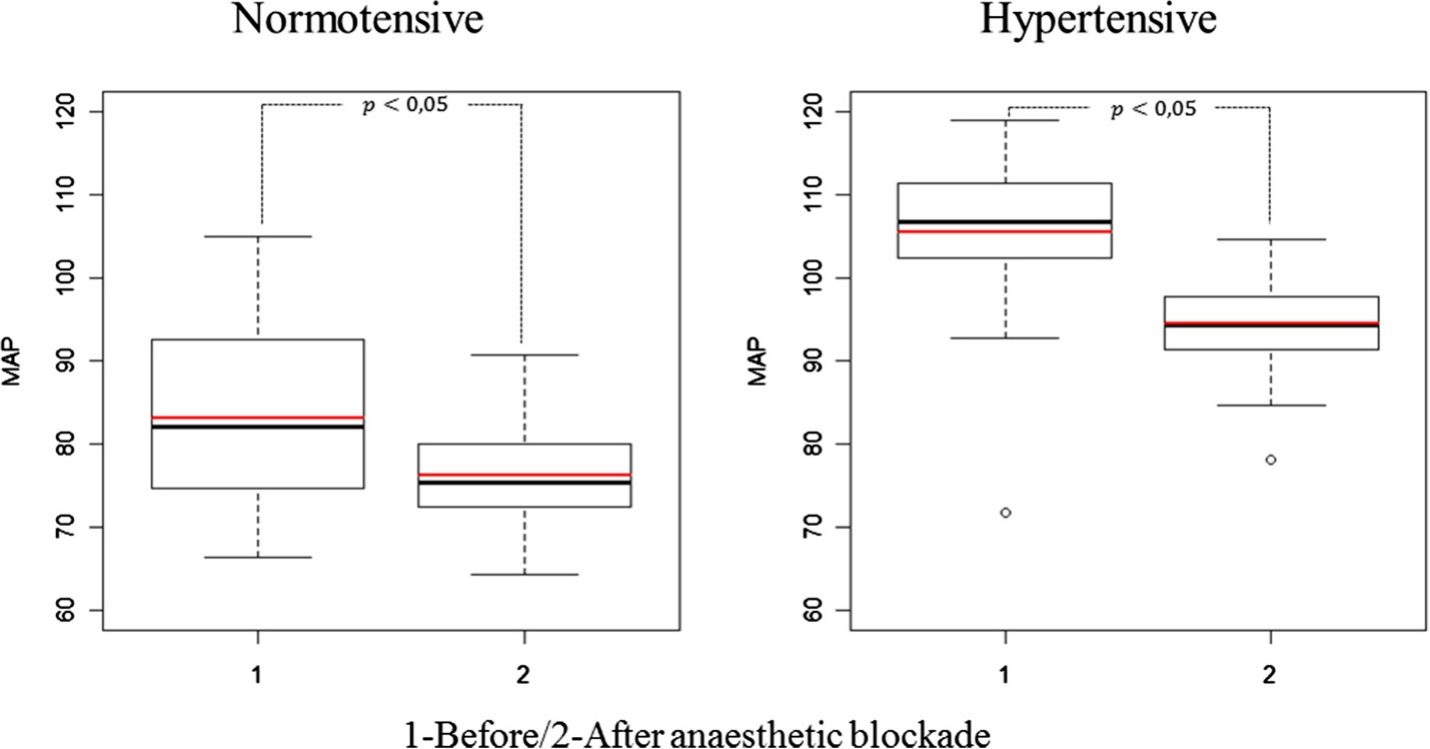
**Boxplots of the observed (black) and predicted (red) MAP values before (1) and after (2) the bupivacaine anaesthetic blockade in the normotensive (left) and hypertensive (right) groups.** The predicted values were determined using the fitted models. The bold horizontal line represents the median while the bottom and top of the box correspond to the 25^th^ and 75^th^ percentiles, respectively; the height of the box is denoted by interquartile range (IQR); the end of the whiskers represent the lowest datum still within 1.5 IQR of the lower quartile, and the highest datum still within 1.5 IQR of the upper quartile. MAP, mean arterial pressure.

Both at the first and second time points, the predicted mean MAP for the HT group was significantly higher than that for the NT group. After blockade, the mean MAP decreased in both groups, with the HT group presenting the highest difference between the two time points (Figure 1). The calculated model for MAP provided expected values for the different combinations of time and hypertensive status (Figure 1 and Table 4) as follows: before anaesthesia, NT group: 83.1 mmHg (95% CI: 81.88–84.5), HT group: 105.5 mmHg (95% CI: 102.2–108.9); and after anaesthesia, NT group: 76.2 mmHg (95% CI: 75.5–77.0), HT group: resp. 94.5 mmHg (95% CI: 92.6–96.5). In particular, the model predicted an 8.3% decrease in the mean MAP in normotensive women and a 10.4% decrease in hypertensive women (p < 0.05).

**Table 4:** **Estimated coefficients and 95% CI of the regression model used to predict the MAP in different variable combinations**

Variables	Coefficient	95% CI
Intercept	83.131	(81.765, 84.498)*
After (*vs* before) anaesthetic blockade	-6.900	(-8.057, -5.743)*
Hypertensive (*vs* normotensive) women	22.414	(18.760, 26.069)*
After anaesthetic blockade and Hypertensive women	-4.130	(-7.224, -1.037)*

*Significant designated at p < 0.05.; CI, confidence interval; MAP, mean arterial pressure.

All regression coefficients were statistically significant (Table 4). Moreover, the single correlation parameter (generally referred to as the intra-class correlation coefficient) was estimated at 0.533 (95% CI: 0.436–0.619), and the variance after anaesthetic blockade was predicted at 57.2% (95% CI: 51.3–63.7%) of the variance before blockade. Statistically significant population mean MAP values were estimated for all of the different time and hypertension combinations (Figure 1).

#### UtA-PI and U-PI model

**Figure 2:**
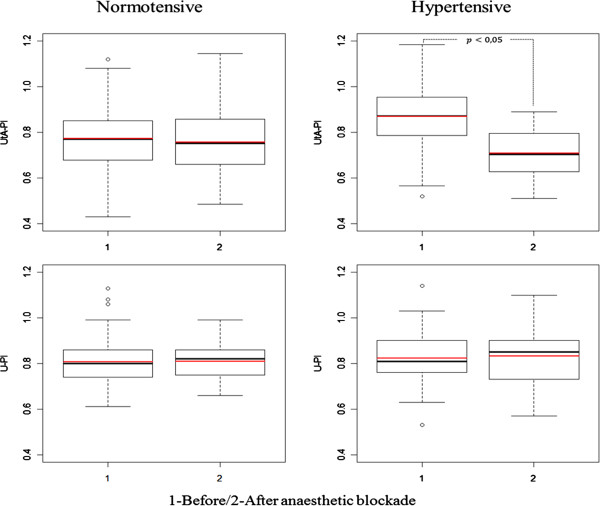
**Boxplots of the observed (black) and predicted (red) UtA-PI and U-PI values before (1) and after (2) the bupivacaine anaesthetic blockade in the normotensive (left) and hypertensive (right) groups.** The predicted values were determined using the fitted models. PI, pulsatility index; UtA, uterine artery; U, umbilical artery.

The model was fitted for both vessels simultaneously. If the process had been done independently for each vessel, the significance level of the conclusions would have been inflated. At the first time point, but not at the second time point, the expected UtA-PI was significantly higher in the HT group than in the NT group. After blockade, there was a statistically significant decrease in the expected UtA-PI only within the HT group (Figure 2). The predicted mean U-PI was not significantly different between the NT and HT groups at any time point, and no significant changes were identified within any of the groups across time (between the first and second time points) (Figure 2). Although all regression coefficients were statistically significant (Table 5) these predictions also accommodated the correlation structure of the model errors. The correlation parameter was estimated at 0.676 (95% CI: 0.623–0.723).

**Table 5:** **Estimated coefficients and correspondent 95% CI of the regression model used to predict the pulsatility index before and after spinal anaesthesia within each artery (UtA or U), adjusted according to the hypertensive status**

Variables	Coefficient	95% CI
Intercept	0.773	(0.757, 0.789)*
After (*vs* Before) anaesthetic blockade	-0.016	(-0.030, -0.002)*
U (*vs* UtA)	0.034	(0.012, 0.056)*
Hypertensive (*vs* Non-Hypertensive) women	0.096	(0.053, 0.139)*
U x Hypertensive women	-0.081	(-0.140, -0.022)*
U x After anaesthetic blockade	0.019	(0.001, 0.037)*
After anaesthetic blockade x Hypertensive women	-0.145	(-0.182, -0.108)*
After anaesthetic blockade x Hypertensive women x U	0.153	(0.104, 0.202)*

*Significance designated at p < 0.05; CI, confidence interval; UtA, uterine artery; U, umbilical artery; PI, pulsatility index.

## Discussion

Maintenance of normotension requires a number of physiologic mechanisms; derangement in these mechanisms may lead to hypertension [26–29]. Hyperactivity of the sympathetic nervous system is one such derangement that has been shown to contribute to hypertension initiation [26], maintenance [28, 29], and progression [27, 29].

Hypertension is a major human disorder and an increasingly important public health issue [30] that extends into pregnancy. Although spinal anaesthesia is the most commonly used method of providing surgical anaesthesia for elective caesarean delivery, spinal hypotension can commonly occur in up to 70% of patients [31]. However, the influence of spinal hypotension on uteroplacental perfusion among women with chronic hypertension has been poorly examined.

To analyse the effect of anaesthetic spinal blockade on uterine and umbilical circulation, normotensive and stage-1 chronic hypertensive pregnant women at term who were undergoing an elective caesarean section were enrolled. Not unexpectedly, we found a statistically significant decrease in MAP after inducing spinal anaesthesia in both groups, most prominently in the chronic HT group. This finding likely reflects the rapid onset of sympathetic blockade [32, 33] and emphasizes the dependence of hypertension on sympathetic intervention, which is distinct from other possible mechanisms such as reduced intravascular volume and left ventricular dysfunction.

It was not our main goal to compare the hypotension incidence between the HT and NT groups, but instead, to study the influence of the decreased MAP during spinal anaesthesia on the uterine artery impedance and to understand the impact on the foetal circulation. Our study is unique as it is the first report evaluating the maternal and foetal circulations in at-term patients with chronic arterial hypertension in the absence of labour, i.e., in the absence of related confounding factors. To that end, Doppler velocimetry was employed because it is a non-invasive tool able to assess the pathophysiological mechanisms underlying foetal [34, 35] and maternal [36–39] perfusion changes.

Because of the difficulty of performing absolute blood flow measurements, most studies have favoured quantitative analysis of flow-velocity waveform change. The PI is the most commonly used index [36, 37] because it describes the shape of the velocity waveform much better than other indexes [36].

There has been concern that during pregnancy, the regulation of pelvic circulation may be disrupted after a sudden decrease in maternal MAP, compromising perfusion to the foetus. This concern was previously addressed in studies performing Doppler velocimetry of umbilical and uterine arteries during epidural analgesia in normal labour patients at term. However, the results were rather conflicting as some authors reported no change in uterine and foetal [40–42] circulations, whereas others found a significant decrease in the resistance of the uterine and umbilical arteries [43]; others even reported an association between the epidural analgesia, maternal hypotension, and increased resistance indexes for the uterine arteries [44, 45]. The variations in reported findings are the likely result of substantially different study conditions. In fact, the previous investigations were performed during labour, had small sample sizes, used less capable ultrasonography systems, or employed epidural analgesia, which causes less sympathetic blockade compared with spinal anaesthesia [46–48].

In this study, UtA-PI was significantly higher in the HT group compared with the NT group before spinal anaesthesia. However, upon establishing spinal anaesthesia, both UtA-PI values decreased to a similar mean, demonstrating that UtA-PI decreased significantly in HT women, compared with NT women. This observation is compelling evidence that chronic hypertensive pregnant women are vulnerable to sympathetic blockade. The findings emphasize the role of the sympathetic nervous system in regulating hypertension and suggest different levels of sympathetic activation in HT patients compared with NT patients [49, 50].

Our findings were obtained from linear and parametric models that satisfied the assumption of errors normality. Nevertheless, the difference in sample size between the hypertensive and the normotensive groups is a study limitation, especially in the PI model, which considers a higher number of parameters than does the MAP model. This limitation must be acknowledged when interpreting the regression coefficients [51]. Fortunately, the number of subjects is reasonably large relative to the number of measurement occasions, which strengthens our study and conclusions. Overall, our numbers were in line with the hypertension prevalence within the respective women age group [52].

### Study limitations and future research

(1) The study comprises uneventful pregnancies and cannot be extrapolated to patients with other forms of hypertensive disease in pregnancy. (2) From the first trimester onwards, HT patients were medicated with 750 mg methyldopa and low-dose acetylsalicylic acid daily, medications that were previously shown not to affect the uterine and umbilical arteries impedance [53–55]. (3) The second Doppler measurement was made just after blockade was achieved, which introduces light variations in time. The Doppler flow evaluation of the right and left uterine and umbilical arteries was performed immediately before performing spinal anaesthesia (first time point) and after blockade was achieved (second time point). Therefore, the time from the first to the second Doppler acquisition was dependent on the time necessary to achieve anaesthetic blockade (~5 minutes). Although not identical for all the patients, the duration was similar among them because only the procedures considered technically easy and uneventful were eligible for the study. In fact, no more than two minutes was required for to measure the waveforms in both the uterine and umbilical arteries (recorded from a free cord loop). Additionally, the measurement sequence was extremely accurate and always performed in the same order: the right uterine artery, the left uterine artery, and finally, the umbilical artery. (4) Serious hypotension requiring VP were excluded, but additional studies will be needed evaluating this subset of patients. (5) There was a sample size imbalance between the hypertensive and the normotensive groups due to elimination of outliers; 16% of the women that were considered for the fitting of the MAP model were hypertensive, while only 17% were in the PI model. While this is adequate for the MAP model, the results inferred from the PI model must be interpreted cautiously. (6) Because we did not assessed neonatal outcomes in detail, no relationship between the vascular indexes and neonatal outcomes in both groups was explored. Thus, the clinical relevance of our findings remains uncertain. (7) There were additional concerns that in the chronic hypertensive population, the reduction of UtA-PI to values equivalent to those in the normal population might actually indicate uterine hypoperfusion and foetal circulation compromise [42, 56], independent of successful compensation against maternal hypotension using VP. This concern is not supported by the current study as there was no change in the U-PI in NT and HT pregnancies, before and after anaesthetic blockade. In contrast, previous studies have shown that in pre-eclamptic women, epidural anaesthesia may help reduce uterine artery vasospasm and may improve intrapartum foetal well-being [18, 19]. However, in pregnant women with chronic arterial hypertension, this benefit has not been demonstrated.

## Conclusions

In conclusion, our results revealed that in normotensive pregnant women at term, the PI of the uterine artery does not change, before and immediately after administering spinal anaesthesia comprising 8–9 mg of hyperbaric bupivacaine (5 mg/mL) and 2–2.5 μg sufentanil (5 μg/mL) intrathecally. However, in chronically hypertensive women with uncomplicated pregnancies, the mean arterial blood pressure decrease was accompanied by a significant reduction in the UtA-PI. Nevertheless, no change in umbilical artery pulsatility index was found, suggesting that despite the UtA-PI drop, the local circulatory mechanisms are apparently activated prevent adverse foetal effects. More research is needed to assess the relationships between U-PI and UtA-PI values with neonatal outcomes among women with hypertensive disorders undergoing spinal anesthesia.

## Electronic supplementary material

Additional file 1: **STROBE Statement—checklist of items that should be included in reports of observational studies.** Description of data: Indicates where each of the recommended items is reported in the manuscript. (DOC 83 KB)

## Abbreviations

BP: Blood pressure
BMI: Body mass index
HT: Hypertensive
ICC: Intraclass correlation coefficients
MAP: Mean arterial pressure
NT: Normotensive
PE: Preeclampsia
PI: Pulsatility index
U: Umbilical artery
UtA: Uterine artery
VP: Vasopressors.
